# Clinical trial design for primary brain tumors: a problem-based framework for neuro-oncology practice

**DOI:** 10.3389/fonc.2026.1936754

**Published:** 2026-09-03

**Authors:** Diego Gómez-Puerto, Jesús Yaringaño, Antonio Di Muzio, Oriol Mirallas, María Vieito

**Affiliations:** 1 Medical Oncology Department, Vall d’Hebron University Hospital and Vall d’Hebron Institute of Oncology, Barcelona, Spain; 2 Department of Investigational Cancer Therapeutics, Division of Cancer Medicine, The University of Texas MD Anderson Cancer Center, Houston, TX, United States

**Keywords:** adaptive platform trial, clinical trial design, external control, glioblastoma, neuro-oncology, patient reported outcome (PRO), primary brain tumor

## Abstract

**Background:**

Primary brain tumor trials may produce an uninterpretable or non-generalizable answer when biological mismatch, inadequate intratumoral exposure, restrictive eligibility, molecular rarity, unstable controls, and disease-specific endpoint ambiguity are addressed separately. The practical problem is not the absence of modern methods, but failure to select and combine them according to the dominant threat to the development decision.

**Methods:**

We performed a practice-facing narrative synthesis of methodological literature, consensus recommendations, regulatory guidance, and published neuro-oncology trials through June 2026. The review focuses primarily on adult trials while identifying areas in which adolescent inclusion or tumor-specific pediatric response frameworks are relevant. For each design problem, we apply four questions: the problem, its failure mode, the design response, and the residual uncertainty.

**Results:**

We propose a problem-based framework that matches each dominant threat to trial validity or feasibility with a prespecified design response. Published trials illustrate why apparently compelling signals can fail at confirmation: ACT IV and INTELLANCE-1 showed no overall-survival benefit despite biomarker selection, and CheckMate 143 showed that a durable minority response signal did not translate into superiority over bevacizumab. Conversely, ROAR and INDIGO demonstrate that strong biological selection, brain-penetrant therapy, and a disease-appropriate population can generate clinically meaningful effects. Quantitative examples show that recurrent-glioblastoma benchmarks vary substantially between historical and contemporary control cohorts. Endpoint hierarchies should combine contemporary Response Assessment in Neuro-Oncology criteria with survival, neurologic function, symptoms, cognition, corticosteroid exposure, seizures, and patient-reported outcomes. We provide a decision tree, disease-specific endpoint map, steroid thresholds, and applied case studies.

**Conclusion:**

A problem-based framework adds value by making the design rationale auditable: each methodological choice is linked to a defined failure mode, quantitative assumptions, and the uncertainty that remains. This approach can improve go/no-go decisions, generalizability, and patient relevance without implying that any single design tool is universally superior.

## 1 Introduction

Primary brain and other central nervous system tumors are uncommon compared with systemic cancers but cause disproportionate morbidity, mortality, neurologic disability, and loss of independence. They comprise more than 100 integrated histomolecular entities in the 2021 World Health Organization classification (1–3). Glioblastoma is the most common malignant primary brain tumor in adults and remains associated with poor outcomes despite maximal safe resection, radiotherapy, and temozolomide-based chemotherapy (4–6). In children and adolescents, brain and other CNS tumors are the most common solid malignancies and a leading cause of cancer-related death, which underscores the importance of age-appropriate trial development (7).

The present review focuses primarily on clinical development in adults with primary brain tumors. Nevertheless, adolescents may appropriately enter selected adult studies when disease biology, formulation, pharmacology, and safety monitoring are shared across age groups; conversely, pediatric-specific entities require pediatric developmental pathways and Response Assessment in Pediatric Neuro-Oncology criteria. The scope also extends beyond glioblastoma to adult IDH-mutant gliomas, meningioma, ependymoma, medulloblastoma, and primary CNS lymphoma when their natural history or response assessment changes the design decision.

The therapeutic landscape is changing. Genomic profiling has identified recurrent actionable alterations, and targeted therapies can produce clinically meaningful activity in selected gliomas, including BRAF V600E-mutant disease (8–11). The phase III INDIGO trial further showed that a brain-penetrant IDH inhibitor can delay progression and the next intervention in carefully selected patients with grade 2 IDH-mutant glioma (12). These successes demonstrate that biological selection matters, but they also expose a central limitation of conventional development: biomarker enrichment, plasma pharmacokinetics, or an early radiographic response does not by itself establish durable patient benefit.

Trial failure in primary brain tumors is rarely attributable to one isolated choice. Model mismatch, inadequate CNS delivery, evolving classification, restrictive eligibility, slow accrual, unstable historical controls, treatment-related imaging change, post-progression therapy, and participant burden can compound one another. A technically sound endpoint cannot rescue a drug that never reaches the tumor; a biologically active drug may appear inactive if enrollment occurs after neurologic decline; and an apparent single-arm signal may reflect something that no longer represents the enrolled population.

This review therefore reframes trial design as an explicit problem-solving exercise. The objective is not to advocate a single preferred design, but to identify the dominant threat to a valid and feasible answer and then select the methodological and operational safeguard that directly addresses that threat. Its novelty is not the invention of randomization, external controls, adaptive methods, perioperative studies, or patient-reported outcomes. Rather, it is the disciplined selection of those tools according to the dominant threat to the decision, with the remaining uncertainty stated before the trial begins. That structure is intended to make protocol rationale, peer review, and go/no-go interpretation more transparent.

### 1.1 Review approach, scope, and four-question framework

This article is a practice-facing narrative review rather than a systematic review or formal guideline. The evidence base comprises peer-reviewed methodological studies, consensus recommendations, representative published clinical trials, public trial protocols when needed to document eligibility thresholds, and regulatory guidance selected for relevance through June 2026. No formal risk-of-bias assessment or quantitative evidence synthesis was performed. Numerical examples are either directly taken from published trials or explicitly identified as illustrative design calculations; they are not original clinical data.

**Figure 1:**
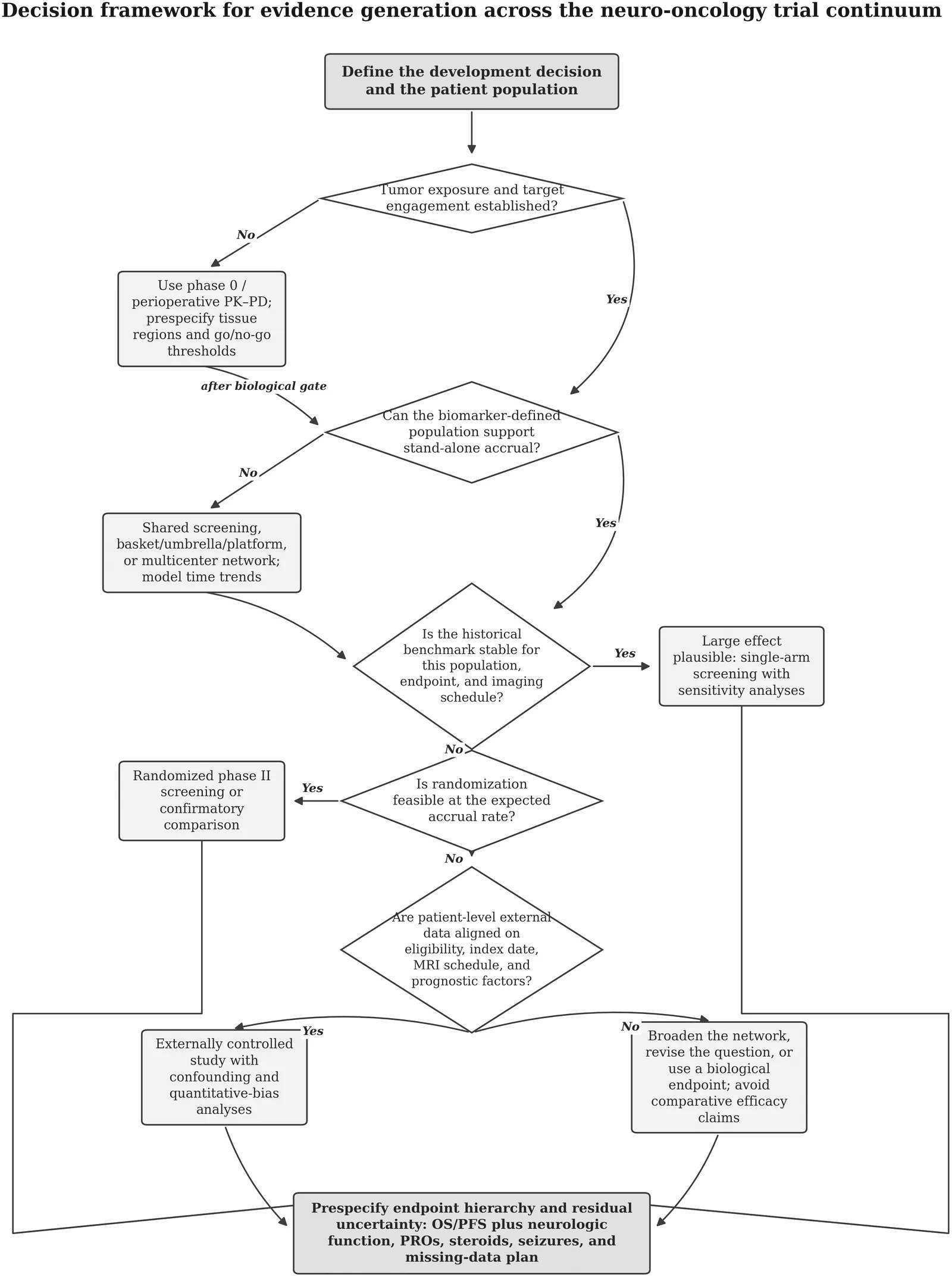
Decision tree for problem-based clinical trial design. The branches prioritize biological gating, accrual feasibility, benchmark stability, and control-data comparability before endpoint selection. At every terminal node, residual uncertainty must be prespecified.

Every major section is organized around four labeled questions: (1) Problem—what biological, statistical, or operational issue most threatens the decision? (2) Failure mode—how could that issue bias the treatment-effect estimate, obscure activity, or prevent enrollment? (3) Design response—which design feature, measurement strategy, or care pathway directly mitigates that failure mode? and (4) Residual uncertainty—what remains unresolved after mitigation? **Table 1** provides the master map. **Figure 1** converts the same logic into a branching decision tree that a protocol team can follow.

**Table 1:** Master problem-based framework for primary brain tumor trial design.

Problem	Failure mode	Design response	Residual uncertainty
Translational mismatch and uncertain CNS exposure	False-positive preclinical signal; false-negative efficacy trial when the compound does not reach or modulate the target.	Hypothesis-matched models; reproducibility; phase 0/perioperative tissue PK–PD; spatial sampling; go/no-go thresholds.	Resection cohorts are selected; short-term target modulation may not predict durable benefit; tissue assays can remain heterogeneous.
Restrictive eligibility and steroid dependence	Slow accrual, center-selection bias, exclusion of neurologically vulnerable patients, and confounded immunotherapy activity.	Mechanism-based exclusions; adolescent/older-adult inclusion when appropriate; explicit dexamethasone ceiling and stability window; stratification and longitudinal steroid capture.	Broader eligibility can increase heterogeneity and safety burden; residual confounding by disease severity persists.
Unstable benchmarks and control selection	A single-arm trial appears positive or negative because case mix, imaging, salvage therapy, or standard care changed.	Contemporary randomization when feasible; target-trial emulation for external controls; benchmark sensitivity analyses.	Randomization may be infeasible in ultra-rare groups; external controls retain unmeasured confounding.
Molecular fragmentation and low accrual	Independent trials do not complete; multiplicity and nonconcurrent controls create fragile inference.	Shared screening; basket/umbrella/platform protocols; prespecified adaptations; simulation; durable governance.	Temporal drift, assay evolution, delayed outcomes, arm competition, and operational complexity remain.
Imaging ambiguity and incomplete patient-benefit assessment	Pseudoprogression/pseudoresponse or steroid effects misclassify activity; radiographic benefit is disconnected from function.	Tumor-specific response criteria; centralized/quality-controlled imaging; endpoint hierarchy; estimands; neurologic, cognitive, seizure, steroid, and PRO outcomes.	No imaging criterion fully distinguishes treatment effect from biology; missing functional/PRO data are informative.
Operational burden and narrow enrollment window	Patients are referred too late, lose eligibility, or cannot travel; data quality varies across sites.	Trial readiness at diagnosis; rapid molecular review; caregiver-inclusive consent; risk-adapted decentralization; regional networks and shared workflows.	Digital, geographic, and caregiver inequities may persist; decentralization can introduce measurement variability.

## 2 Translational mismatch and intratumoral exposure

### 2.1 Problem

No preclinical model reproduces the full spatial, cellular, vascular, immune, and evolutionary complexity of a human brain tumor. Genetically engineered mouse models can test defined drivers in an intact immune system; orthotopic patient-derived xenografts preserve selected tumor-intrinsic features and an anatomic brain environment; and patient-derived organoids permit higher-throughput perturbation and co-culture studies. The relevant question is not which model is globally “best,” but whether the model contains the biology required by the therapeutic hypothesis (13–15).

### 2.2 Failure mode

Model mismatch can generate both false-positive and false-negative translation. A therapy may look active in a model with an absent blood-brain barrier, homogeneous target expression, or limited myeloid suppression, then fail clinically. Conversely, a valid target may be abandoned when the molecule has inadequate unbound intratumoral exposure. Published phase III trials illustrate that biomarker selection alone is not sufficient. ACT IV randomized 745 patients with EGFRvIII-positive newly diagnosed glioblastoma and reported virtually identical median overall survival, 20.1 versus 20.0 months, despite a biologically specific vaccine strategy (16). INTELLANCE-1 randomized 639 patients with EGFR-amplified newly diagnosed glioblastoma. Median overall survival was 18.9 versus 18.7 months, although progression-free survival modestly favored depatuxizumab mafodotin (17). These were real comparative failures, not hypothetical scenarios.

The interpretation should not be that EGFR biology is irrelevant. Rather, antigen heterogeneity and loss, payload delivery, pharmacodynamic adequacy, and the relation between early imaging and survival remain competing explanations. CheckMate 143 provides a related example: nivolumab produced fewer responses than bevacizumab and did not improve median overall survival (9.8 versus 10.0 months), yet responses in a minority were durable. Without paired tissue and mechanistic sampling, a negative population-level result cannot distinguish inadequate immune activation from an invalid target or an unsuitable clinical state (18, 19). A summary of published trial examples illustrating these distinct failure modes and their associated design lessons is provided in **Table 2**.

**Table 2:** Published trial examples used to distinguish failure modes from hypothetical scenarios.

Trial and population	Design and key result	Problem exposed	Design lesson
ACT IV; EGFRvIII-positive newly diagnosed glioblastoma	Phase III, n=745; median OS 20.1 vs 20.0 months.	Biomarker selection did not overcome antigen heterogeneity/loss or other translational limitations.	Confirm spatial and longitudinal target persistence; integrate reverse translation.
INTELLANCE-1; EGFR-amplified newly diagnosed glioblastoma	Phase III, n=639; median OS 18.9 vs 18.7 months; modest PFS difference.	Target expression and early disease control did not translate into OS benefit.	Demonstrate payload exposure/PD and prespecify endpoint hierarchy.
CheckMate 143; recurrent glioblastoma	Phase III, n=369; median OS 9.8 vs 10.0 months; ORR 7.8% vs 23.1%.	Population-level failure despite durable responses in a minority.	Collect tissue, steroid exposure, and immune-state markers; avoid equating a negative trial with target invalidity.
ROAR; BRAF V600E-mutant high-grade glioma	Basket phase II; high-grade cohort n=45; ORR 33%.	Rare, strongly selected biology can yield a large signal.	Basket development is credible when target, drug exposure, and effect size are compelling.
INDIGO; grade 2 IDH-mutant glioma	Phase III, n=331; median PFS 27.7 vs 11.1 months; HR 0.39.	Long natural history requires a disease-state-specific endpoint and population.	Match the intervention to an observable, clinically relevant disease window.
GBM AGILE regorafenib; newly diagnosed and recurrent glioblastoma	Bayesian platform report, 2026; no superiority over control in either setting.	Adaptive infrastructure cannot rescue an inactive agent and must manage time trends/nonconcurrent controls.	Publish operating characteristics and treatment-arm results, including negative results.

Therapies examples to show how the same four questions can separate a molecule problem, a delivery problem, a population problem, and an endpoint/control problem. This is the principal distinction between a catalog of designs and a failure-oriented analysis.

### 2.3 Design response

A credible translational package should document molecular identity, clonal stability, growth pattern, relevant microenvironmental features, assay reproducibility, and exposure at clinically plausible doses. Positive findings are more persuasive when reproduced across complementary models and when negative or heterogeneous findings are reported. Reverse translation should be planned before the first efficacy trial: on-treatment tumor, blood, and when appropriate cerebrospinal fluid can determine whether failure resulted from inadequate exposure, absent target engagement, adaptive signaling, clonal evolution, or immunosuppression.

Central nervous system delivery should be demonstrated before resource-intensive efficacy testing when tissue access is feasible. Plasma pharmacokinetics do not establish unbound exposure in enhancing and nonenhancing tumor. Phase 0 and perioperative window studies can quantify tissue pharmacokinetics, target engagement, and downstream modulation in patients already scheduled for resection (20, 21). Protocols should prespecify tissue regions, dose-to-surgery interval, assay validation, treatment of necrotic tissue, and a decision matrix. For example: adequate exposure plus target modulation supports efficacy development; adequate exposure without modulation challenges the mechanism or dose; inadequate exposure challenges the molecule rather than the target.

### 2.4 Residual uncertainty

Perioperative studies preferentially enroll patients with resectable disease, a short exposure window may not predict durable clinical benefit, and a sampled enhancing region may overstate delivery to infiltrative tumor. Spatially annotated sampling, matched plasma and tissue, and central laboratory quality control reduce but do not eliminate these limitations. The design conclusion should therefore be probabilistic: biological gating can prevent an uninterpretable phase II trial, but it cannot prove later efficacy.

## 3 Representative enrollment, corticosteroids, and patient-centered conduct

### 3.1 Problem

Enrollment is limited by low awareness, neurologic and cognitive impairment, caregiver burden, distance from specialist centers, socioeconomic constraints, and a narrow therapeutic window at recurrence (22–25). Restrictive criteria may be particularly consequential in adolescents, older adults, patients with controlled seizures, and patients requiring low-dose corticosteroids. Participants at tertiary centers can differ systematically from the population in routine practice, and delayed referral selects for unusually stable disease.

### 3.2 Failure mode

Unnecessary tissue requirements, prolonged washout, broad exclusions for prior therapy, and arbitrary age cutoffs reduce accrual and external validity (26). Steroid criteria create a specific tension. Dexamethasone can suppress immune function and is associated with worse outcomes in several immunotherapy settings, but excluding every patient requiring steroids selects for lower tumor burden and better neurologic status. The treatment-effect estimate can therefore be biased even when the biological rationale for restricting steroids is sound (27).

### 3.3 Design response

Eligibility should be justified by mechanism, safety, or interpretability. Adolescents may enter selected adult trials when the integrated diagnosis, target, formulation, and monitoring are shared; older adults should be characterized by performance status, comorbidity, cognition, medication burden, and geriatric assessment rather than age alone (28–30). A screening log should record each exclusion reason and selected baseline features among nonenrolled patients so that representativeness is measurable.

Steroid rules should be numerical and trial-specific. Published and publicly available neuro-oncology immunotherapy protocols commonly require a stable or decreasing dexamethasone dose for at least 5–7 days and cap baseline exposure at 2–4 mg/day. The perioperative pembrolizumab protocol NCT02337686 used no more than 2 mg/day for at least 5 days, while several pragmatic or combination immunotherapy studies have allowed up to 4 mg/day (31, 32). These values should be treated as examples, not a universal standard. A proof-of-mechanism immunotherapy study may reasonably use ≤2 mg/day, stable/decreasing for ≥5 days; a broader efficacy trial may allow ≤4 mg/day, stratify by 0 versus >0–4 mg/day, and record dose as a time-varying covariate. Trials not dependent on immune activation may use wider limits based on safety and imaging interpretability.

Consent must be integrated into routine counseling, delivered in plain language, and repeated when necessary. Capacity assessment, legally authorized representatives, and caregiver involvement should preserve the participant’s voice. Decentralization is not equivalent to reduced oversight. Hybrid conduct can use local laboratories, quality-controlled local MRI, telehealth for suitable visits, home nursing, and shipment of eligible oral agents, while protocols must define which procedures may occur remotely, how urgent neurologic events are assessed, how source data and imaging quality are verified (33–35).

### 3.4 Residual uncertainty

Broader eligibility improves representativeness but can increase heterogeneity and safety burden. Steroid dose remains a marker of disease severity even after stratification, and time-varying escalation may be both a confounder and an outcome. Decentralization can reduce travel while worsening digital or caregiver inequity and introducing measurement variability. These effects have to be reported rather than assumed to cancel out.

## 4 Credible phase II decisions, randomization, and external controls

### 4.1 Problem

Phase II trials need to support an explicit development decision: stop, refine the dose or population, or proceed to confirmation. Single-arm designs efficiently screen for a large effect when natural history is stable and the endpoint is reliable. In primary brain tumors, diagnostic drift, molecular reclassification, imaging schedules, corticosteroids, local therapy, and subsequent treatment can make historical benchmarks unstable (36–41).

### 4.2 Failure mode

Recurrent glioblastoma shows the benchmark problem directly. Historical cytotoxic datasets supported a 6-month progression-free survival benchmark near 15% (42). In the BRAIN bevacizumab cohort, however, PFS6 was 42.6% and median overall survival 9.2 months (43). Later randomized control groups varied again: lomustine median overall survival was 8.6 months in EORTC 26101, 5.6 months in REGOMA, and bevacizumab median overall survival was 10.0 months in CheckMate 143 (18, 44, 45). In the 2026 GBM AGILE analysis, recurrent-disease control median overall survival was approximately 9.7 months (46). These populations, imaging rules, treatments, and eras are not interchangeable. The historical evolution and variability of these recurrent glioblastoma benchmarks across different eras and study designs are detailed in **Table 3**.

**Table 3:** Illustration of benchmark variability in recurrent glioblastoma.

Source/era	Control or treatment cohort	Key benchmark	Why direct reuse may fail
Historical NABTC datasets (published 2008)	Multiple recurrent high-grade glioma phase II cohorts	PFS6 benchmark approximately 15% for recurrent glioblastoma.	Preludes current molecular classification, imaging practice, bevacizumab exposure, and contemporary salvage care.
BRAIN (2009)	Bevacizumab alone, n=85	PFS6 42.6%; median OS 9.2 months.	Anti-VEGF pseudoresponse and a noncomparative selected cohort inflate incompatibility with older cytotoxic benchmarks.
EORTC 26101 (2017)	Lomustine control	Median PFS 1.5 months; median OS 8.6 months.	First recurrence, trial eligibility, and central imaging differ from many real-world or later-line datasets.
REGOMA (2019)	Lomustine control, n=60	Median OS 5.6 months.	Small randomized phase II control with different geography, eligibility, and subsequent therapy.
CheckMate 143 (2020)	Bevacizumab control, n=185	Median PFS 3.5 months; median OS 10.0 months; ORR 23.1%.	Comparator mechanism, response criteria, and steroid/case mix differ from lomustine controls.
GBM AGILE report (2026)	Contemporary platform control, recurrent disease	Median OS approximately 9.7 months.	Nonconcurrent control use and platform-era changes require explicit time adjustment and governance.

Cases showing why a protocol must justify the chosen benchmark by molecular definition, line of therapy, eligibility, index date, imaging schedule, and access to post-progression therapy. A sensitivity analysis should repeat the operating characteristics across plausible control rates rather than use a single historical point estimate.

### 4.3 Design response

Randomized phase II screening designs provide contemporary control and protect against measured and unmeasured prognostic imbalance. They are especially useful when the expected effect is modest, the natural history is uncertain, or the endpoint is susceptible to assessment bias. Their purpose can be estimation and selection rather than definitive hypothesis testing.

A worked calculation makes the trade-off concrete. Consider 1:1 randomization, a one-sided alpha of 0.10, 80% power, and a target hazard ratio of 0.70. A standard log-rank approximation requires about 142 events. If 90% of participants are expected to experience the event, approximately 158 participants are required. At four participants per month, accrual alone would take about 40 months; at eight per month, about 20 months. By contrast, an exact single-arm PFS6 design testing 15% versus 30% with one-sided alpha ≤0.10 and power ≥0.80 can use n=37 and declare activity with at least 9 progression-free patients at 6 months (actual alpha approximately 0.092; power approximately 0.824). The efficiency gain is substantial, but if the true contemporary control PFS6 is 25%-30%, the single-arm conclusion becomes misleading. These calculations are illustrative and have to be replaced by protocol-specific assumptions, nonproportional-hazard checks, dropout, and assessment timing.

External controls are appropriate when randomization is infeasible or ethically problematic, but an eligibility-matched database is insufficient. The target population, index date, treatment setting, outcome definitions, MRI schedule, follow-up, prognostic variables, and subsequent therapy should emulate the prospective trial. The analysis plan ought to prespecify confounding adjustment, missing-data methods, overlap diagnostics, sensitivity analyses, and quantitative-bias or negative-control analyses when feasible (39–41).

### 4.4 Residual uncertainty

Randomization can still fail through inadequate accrual, treatment crossover, nonadherence, or insensitive endpoints. External controls cannot remove unmeasured confounding or guarantee comparable imaging and supportive care. Single-arm designs remain reasonable for very large effects in highly selected molecular disease, but the protocol should state the minimum effect size that would remain convincing under less favorable benchmark assumptions.

## 5 Molecular fragmentation and adaptive or master-protocol designs

### 5.1 Problem

Molecular classification improves biological coherence while dividing already uncommon diseases into subgroups that may not support independent trials. In a 2026 multicenter real-world sequencing series, 541 patients with glioma were profiled; among 409 glioblastomas, approximately 9% had tier 1 or 2 alterations involving IDH1/2, BRAF V600E, FGFR1-3, or NTRK, and only 10.2% of the overall cohort received molecularly matched therapy (10). Even this aggregate conceals alterations with individual prevalence around 1%–3% (47).

### 5.2 Failure mode

The rarity problem can be expressed operationally. A center screening 150 adult glioma patients per year would identify approximately 14 patients annually if 9% carry a high-level actionable alteration. For a single alteration present in 1%-3%, only about 2–5 molecularly eligible patients would be found before excluding for disease setting, performance status, organ function, prior therapy, geography, or trial availability. After those filters, a center may enroll only one or two patients per year. A stand-alone 40-patient study could therefore require an unrealistic number of centers and years.

### 5.3 Design response

Umbrella, basket, and platform protocols can share screening, biomarker, imaging, control, and governance infrastructure (48–52). INSIGhT, GBM AGILE, and N2M2 illustrate different approaches to continuous learning and real-time molecular assignment. Adaptations may include futility stopping, sample-size modification, arm selection, or seamless transition, but each must be specified in the protocol and statistical analysis plan. Simulation should address type I error or posterior thresholds, multiplicity, delayed outcomes, arm entry and exit, control allocation, nonconcurrent controls, overrunning enrollment, and changes in standard care (40, 53, 54).

The updated EANO molecular-testing guideline emphasizes that routine sequencing should distinguish diagnostic necessity from therapeutic actionability and that evidence differs substantially by target (60). A master protocol should therefore avoid treating every genomic alteration as equally validated. Entry criteria can use tiered evidence, require pathway-level confirmation, and incorporate a biological gate such as intratumoral exposure for agents with uncertain CNS penetration.

### 5.4 Residual uncertainty

Platforms do not create patients or active drugs. They may intensify competition among arms, rely on evolving assays, and become vulnerable to temporal drift and nonconcurrent-control bias. Response-adaptive randomization also requires caution when outcomes are delayed or when early endpoints are weak surrogates for survival. The 2026 GBM AGILE regorafenib report is informative precisely because a sophisticated Bayesian platform did not show superiority in newly diagnosed or recurrent disease (46). Transparent negative results, operating characteristics, and dates of control contribution are essential to preserve credibility.

## 6 Endpoint strategy across distinct primary CNS tumors

### 6.1 Problem

An endpoint can be reliable yet answer the wrong clinical question. Overall survival is definitive but delayed and influenced by crossover and post-progression treatment. Progression-free survival, PFS6, and objective response provide earlier signals but are vulnerable to pseudoprogression, pseudoresponse, anti-VEGF effects, steroid change, and imaging frequency (41, 55). The problem is not uniform across primary CNS tumors: adult glioma, meningioma, ependymoma, medulloblastoma, and primary CNS lymphoma have different dissemination patterns, growth rates, imaging characteristics, and clinically meaningful outcomes.

### 6.2 Failure mode

Using RANO 2.0 as a universal CNS-tumor solution would be incorrect. RANO 2.0 provides a framework for adult high- and low-grade gliomas, with standardized baseline selection, confirmation rules, and integration of clinical status and corticosteroids (56). Meningioma trials require bidimensional or volumetric assessment adapted to often slow and irregular growth and may use PFS6 standards stratified by grade (57). Medulloblastoma and ependymoma require brain and spine MRI, cerebrospinal fluid assessment when appropriate, and attention to leptomeningeal disease (58, 59). Primary CNS lymphoma response assessment must account for corticosteroid sensitivity, diffusion, enhancement, ocular disease, and CSF involvement (60).

### 6.3 Design response

Endpoint choice should follow the trial objective and mechanism. Perioperative studies may prioritize tissue exposure and target modulation. A randomized phase II study may use progression-free survival with rigorous image acquisition and central review. A registration trial will generally require overall survival or another endpoint supported by disease setting, magnitude and durability of effect, and regulatory precedent. The protocol must define population, treatment condition, outcome, summary measure, and handling of intercurrent events such as discontinuation, crossover, resection, rescue therapy, and death before assessment (61).

Radiographic stability is insufficient when neurologic function worsens, cognition declines, corticosteroids increase, seizures become more frequent, or quality of life deteriorates. Conversely, a modest imaging effect may matter when accompanied by steroid reduction, preserved independence, symptom improvement, seizure control, or delayed next treatment. NANO and validated patient-reported instruments such as the MDASI-BT can complement survival and imaging (62–64). A concise prespecified battery is preferable to overlapping questionnaires that increase missingness.

### 6.4 Residual uncertainty

No criterion fully separates treatment effect from natural history, treatment-related change, and supportive care. Missing neurologic and patient-reported data are rarely random because deterioration, hospitalization, cognitive decline, and caregiver strain prevent assessment. Protocols should define windows, accessible modes, proxy rules when justified, post-discontinuation collection, and pattern-mixture or tipping-point sensitivity analyses. Completion rates and reasons for missingness should be reported by arm and time point.

## 7 Operational burden, trial readiness, and research networks

### 7.1 Problem

Trial design is also a model of care delivery. Tissue acquisition, molecular testing, baseline neurologic examination, steroid trajectory, antiseizure therapy, imaging intervals, and referral pathways are usually established before recurrence. If trial readiness begins only after progression, rapid neurologic decline, tissue depletion, washout requirements, or delayed sequencing may eliminate eligibility.

### 7.2 Failure mode

A statistically efficient protocol can fail through slow molecular turnaround, inconsistent MRI, limited caregiver support, or geographic concentration. Restrictive site selection may improve data quality while reducing access. Conversely, decentralization without quality control may introduce endpoint noise and unsafe delays in evaluating seizures, edema, or neurologic decline.

### 7.3 Design response

The multidisciplinary tumor board is a practical integration point. At diagnosis and each progression, the team should document integrated diagnosis, molecular profile, MGMT promoter methylation when relevant, extent of resection, performance and neurologic status, steroid dose and trajectory, seizures, comorbidity, caregiver support, and proximity to trial resources. Early identification can preserve tissue and prevent avoidable washout.

Academic and cooperative networks are essential for questions unlikely to attract commercial investment, including treatment sequencing, supportive care, imaging schedules, steroid-sparing strategies, and optimization of approved therapies (65). Shared screening logs, common molecular and imaging workflows, centralized statistical support, and regional referral agreements reduce duplication. Under the European Clinical Trials Regulation, low-intervention pathways may facilitate pragmatic comparative studies when legal criteria are met, but lower operational burden should not weaken protocol clarity or data integrity (66).

### 7.4 Residual uncertainty

Networks require durable funding, governance, and equitable referral. Hybrid visits may transfer work to families or community clinicians, and local imaging may vary despite credentialing. Protocols should prospectively measure travel, time, caregiver burden, screen failure, and data-query rates to determine whether operational adaptations improve participation and data quality.

## 8 Worked applied case studies of the four-question framework

### 8.1 Case 1: recurrent glioblastoma immunotherapy with an expected modest effect

Problem: The contemporary PFS and OS is unstable, corticosteroids may modify both prognosis and immune activity, and early imaging can be misleading.

Failure mode: A 35-patient single-arm trial could appear active because it enrolls steroid-free patients with smaller tumors and compares them with an older 15% PFS6 benchmark. Conversely, delayed immune responses could be classified as progression.

Design response: Use a randomized phase II screening design with stratification by baseline dexamethasone (0 vs >0–4 mg/day), MGMT status, and first versus later recurrence; require stable/decreasing steroids for at least 5 days; standardize MRI and use mechanism-appropriate confirmation; collect tissue/immune correlates in a surgical subset; include NANO, steroid trajectory, seizures, and PROs.

Residual uncertainty: The trial may still be underpowered for a biomarker-defined responder subset, and post-progression bevacizumab can affect both symptoms and survival. The protocol should state that exploratory immune signatures are hypothesis-generating unless prospectively validated.

### 8.2 Case 2: FGFR-altered glioma in an ultra-rare molecular subgroup

Problem: A single alteration may occur in only 1%-3% of screened gliomas, and CNS exposure of the selected inhibitor may be uncertain.

Failure mode: A stand-alone trial accrues too slowly, while a histology-agnostic basket may pool tumors with different fusion partners, co-alterations, and drug delivery. An apparent response in enhancing tumor may not represent infiltrative disease.

Design response: Use multicenter shared screening or a basket/platform cohort with molecular central review, prespecified alteration classes, and a perioperative PK–PD cohort when resection is planned. A single-arm cohort may be acceptable if the expected response is large and durable, but use exact confidence intervals and compare results with carefully aligned patient-level natural-history data rather than a generic glioma control.

Residual uncertainty: Very small samples limit partner-specific inference, resistance mechanisms may differ, and resection-based PK data remain selected. Confirmatory evidence may require pooling across coordinated protocols rather than one conventional phase III trial.

### 8.3 Case 3: systemic therapy for progressive meningioma

Problem: Growth is often slow and irregular, prior surgery/radiotherapy is heterogeneous, and objective response is uncommon even when growth is slowed.

Failure mode: A short response-rate study incorrectly declares inactivity, or a single-arm PFS6 study uses a benchmark that mixes WHO grades and prior treatments.

Design response: Require documented pre-enrollment growth over a defined interval; stratify by WHO grade and prior radiotherapy; apply RANO meningioma measurement; select PFS6 or growth-rate change as the phase II decision endpoint with symptoms, neurologic function, steroid use, and quality of life; randomize when the expected effect is modest and a feasible comparator exists.

Residual uncertainty: Volumetric methods and SSTR PET may improve measurement but are not fully standardized as efficacy endpoints, and prolonged follow-up is needed to connect growth control with patient benefit.

## 9 Design trade-offs and clinic-ready implementation

The framework does not prescribe one design. **Table 4** compares common phase II and adaptive structures; **Table 5** links endpoint domains to safeguards; **Table 6** translates them into routine neuro-oncology workflows. These tables should be read horizontally: the strength of each tool is conditional on the failure mode, and the final column identifies where implementation can still fail.

**Table 4:** Practical considerations for selected phase II and adaptive designs.

Design	Strengths	Principal limitations/residual uncertainty	Best suited for
Single-arm phase II	Fast; smaller sample; efficient for futility or large-effect screening.	Depends on a stable benchmark; vulnerable to case mix, diagnostic drift, assessment schedule, and subsequent therapy.	Well-characterized natural history and objective endpoint; rare subgroup with a compelling expected effect.
Randomized phase II screening	Contemporary control; internal treatment-effect estimate; protects against prognostic imbalance.	More participants, cost, and accrual time; imprecise if screened as a small phase II.	Unstable benchmark, modest expected effect, or assessment-sensitive endpoint.
Externally controlled study	Conserves participants; uses patient-level historical/real-world data.	Unmeasured confounding, index-date bias, missing prognostic variables, temporal drift, and outcome misalignment.	Randomization infeasible; deeply harmonized data; large expected effect.
Seamless phase II/III	Reduces start-up delay and may carry eligible participants into confirmation.	Requires transition rules, multiplicity control, consistent endpoint definition, and early regulatory alignment.	Promising regimen with a credible intermediate endpoint and feasible confirmatory pathway.
Adaptive platform/master protocol	Shares screening/control/infrastructure; permits arm entry and exit.	Temporal drift, nonconcurrent controls, delayed outcomes, arm competition, simulation and governance burden.	Repeated hypotheses and rare molecular subtypes within a sustained network.

**Table 5:** Endpoint strategy across primary brain tumor trial phases.

Endpoint domain	Advantages	Principal risks	Recommended safeguards
Intratumoral PK/PD and target engagement	Confirms tumor exposure and biological modulation before efficacy testing.	Sampling bias, spatial heterogeneity, assay variability, perioperative selection.	Matched plasma/tissue; validated assays; spatial annotation; prespecified decision matrix.
Overall survival	Direct clinical relevance and objective ascertainment.	Long follow-up; crossover and subsequent therapy.	Stratification; prospective post-progression capture; estimand and sensitivity analyses.
PFS, PFS6, ORR	Earlier readout; potentially smaller trials.	Pseudoprogression/pseudoresponse; MRI timing; steroids and anti-VEGF effects.	Tumor-specific criteria; standardized MRI; central review; clinical/steroid documentation.
PFS2/time to next intervention	Captures durability and treatment sequencing.	Influenced by subsequent treatment and inconsistent ascertainment.	Clear definitions; prospective collection of intercurrent events and subsequent therapy.
Neurologic and patient-reported outcomes	Measures function, symptoms, cognition, independence, and quality of life.	Informative missingness, instrument heterogeneity, participant burden.	Validated concise battery; accessible modes; proxy plan; longitudinal missing-data strategy.
Steroid and seizure outcomes	Directly reflects neurologic burden and treatment effects.	Confounded by clinician practice and disease severity.	Dose-equivalent conversion; time-varying capture; protocolized escalation/taper where feasible; seizure diary and medication changes.

**Table 6:** Translating trial-design solutions into neuro-oncology practice.

Practice domain	Common barrier	Trial-design adaptation	Practical implementation
Diagnosis and tissue pathway	Limited tissue, delayed molecular results, evolving WHO entities.	Plan biomarker and tissue strategy before activation.	Coordinate neurosurgery, neuropathology, molecular board, and trial office; reflex testing; tissue inventory.
Consultation and consent	Low awareness, cognitive impairment, caregiver burden.	Embed trial education and capacity assessment in standard counseling.	Plain-language material; caregiver-inclusive process; repeat discussion; early support referral.
Eligibility and prescreening	Age cutoffs, steroid restrictions, washout, broad prior-therapy exclusions.	Justify each criterion by mechanism, safety, or interpretability.	Checklist for function, neurologic deficits, dexamethasone, antiseizure drugs, organ function, and timing.
Treatment delivery	Travel, disability, fatigue, seizures, caregiver burden.	Risk-adapted decentralized elements.	Local labs/MRI after credentialing; suitable telehealth; home nursing; community partnerships.
Imaging assessment	Pseudoprogression, variable MRI timing, steroid effects.	Tumor-specific criteria and clinical-context capture.	Record steroid dose, neurologic status, surgery/radiotherapy/anti-VEGF timing; central review when decisive.
Outcome assessment	OS delayed; PFS may not reflect benefit.	Prespecified hierarchy and estimand.	Collect OS, PFS/PFS2, neurologic function, cognition, PROs, seizures, steroids, and subsequent therapy.
Recurrent disease	Rapid decline and narrow enrollment window.	Rapid matching and rescue pathways.	Identify trials before progression; minimize washout; accelerated molecular review; off-protocol rescue plan.
Network development	Centralized access, fragmented workflows, limited funding.	Cooperative, low-intervention, and platform approaches.	Regional referrals; shared screening logs; common molecular/imaging workflows; centralized statistics.

## 10 Discussion

### 10.1 What the framework adds to contemporary practice

Many methods discussed in this review are already incorporated into contemporary trials. The practical contribution is therefore not novelty at the level of individual tools. It is the requirement that a protocol name the threat being mitigated, quantify the assumptions where possible, and state the uncertainty that remains. This prevents method accumulation without rationale, for example, adding an external control because it is available, an adaptive feature because it is fashionable, or multiple patient-reported instruments without a defined endpoints.

The framework also makes interacting problems visible. Restrictive steroid criteria can improve immunologic interpretability while worsening accrual and generalizability. A molecularly precise cohort may improve effect size while destabilizing its historical benchmark. A decentralized MRI pathway may improve access while increasing measurement variance. These are not reasons to avoid the tools; they are reasons to identify the trade-off prospectively and monitor it.

### 10.2 Key controversies

Randomization offers the strongest protection against confounding but may be infeasible in ultra-rare subsets. External controls conserve participants but exchange randomization for assumptions about comparability. Broad eligibility improves representativeness but can dilute a mechanism-specific effect; narrow eligibility improves biological coherence while limiting applicability. Adaptive and platform designs provide efficiency only when data flow, endpoint timing, and governance are reliable. RANO-based response improves standardization but cannot substitute for how a patient feels, functions, or survives.

Adaptive and platform designs provide efficiency only when the endpoint is timely, data flow is reliable, and the infrastructure can sustain complex governance. A sophisticated statistical model cannot compensate for delayed molecular testing, inconsistent magnetic resonance imaging, or insufficient accrual. Similarly, RANO-based response improves standardization but does not resolve the fundamental distinction between radiographic change and how a patient feels, functions, or survives.

These trade-offs support a layered evidence strategy. Early development should establish exposure and target engagement when uncertainty is material. Phase II should use a control strategy proportional to benchmark stability and expected effect size. Confirmatory trials should define estimands and endpoint hierarchies before enrollment. Across phases, neurologic function, symptoms, treatment burden, and access should be treated as design variables.

### 10.3 Research priorities

Priority areas include harmonized reporting of tissue exposure and spatial sampling; prospective validation of external-control adjustment; public platform simulations and time-trend analyses; screening logs that measure representativeness; clinically interpretable cognition, communication, independence, seizure, steroid, and time-at-home outcomes; and evaluation of decentralized procedures for safety, data quality, and equity. Cross-tumor application is also needed because the dominant threat in an indolent IDH-mutant glioma, progressive meningioma, disseminated medulloblastoma, or primary CNS lymphoma differs from recurrent glioblastoma.

### 10.4 Strengths and limitations of this review

The review integrates biological, statistical, operational, and patient-centered considerations and applies them through a consistent four-question structure. It adds quantitative examples, published trial comparisons, tumor-specific response frameworks, and worked applied case studies. It remains a narrative review subject to selection bias and does not provide a systematic search, formal evidence grading, or quantitative meta-analysis. The calculations are illustrative, not protocol-ready sample-size determinations. Examples still emphasize glioma because it has the largest adult trial literature, and recommendations for pediatric entities or ultra-rare adult tumors require disease-specific expert and regulatory input. Public protocol eligibility criteria can document practice but do not establish an evidence-based universal steroid threshold. Regulatory expectations evolve and should be discussed prospectively with relevant agencies.

## 11 Conclusions

Clinical trial design in primary brain tumors should move beyond a generic phase-based template toward an auditable problem-based strategy. The protocol team should identify the dominant threat, describe how it would create failure, choose the response that directly mitigates it, and state the residual uncertainty. Real trial results show why this distinction matters: biological selection may fail without durable target expression or effective delivery; adaptive infrastructure may still yield a negative answer; and a single-arm result can change meaning when the benchmark changes.

The most actionable improvements are hypothesis-matched and reproducible models; perioperative PK–PD studies when delivery is uncertain; mechanism-based eligibility with numerical steroid rules; contemporary randomization when benchmarks are unstable; disciplined emulation and bias analysis for external controls; transparent adaptive/master-protocol governance; tumor-specific response criteria; and endpoint hierarchies that integrate survival, imaging, neurologic function, symptoms, cognition, corticosteroids, seizures, and quality of life. Embedding the same logic in routine workflows can improve feasibility, generalizability, and the clinical relevance of neuro-oncology trials.
